# Insights on embodiment induced by visuo-tactile stimulation during robotic telepresence

**DOI:** 10.1038/s41598-021-02091-8

**Published:** 2021-11-22

**Authors:** D. Farizon, P. F. Dominey, J. Ventre-Dominey

**Affiliations:** INSERM UMR1093-CAPS, Université Bourgogne Franche-Comté, UFR des Sciences du Sport, 21000 Dijon, France

**Keywords:** Neuroscience, Psychology

## Abstract

Using a simple neuroscience-inspired procedure to beam human subjects into robots, we previously demonstrated by visuo-motor manipulations that embodiment into a robot can enhance the acceptability and closeness felt towards the robot. In that study, the feelings of likeability and closeness toward the robot were significantly related to the sense of agency, independently of the sensations of enfacement and location. Here, using the same paradigm we investigated the effect of a purely sensory manipulation on the sense of robotic embodiment associated to social cognition. Wearing a head-mounted display, participants saw the visual scene captured from the robot eyes. By positioning a mirror in front of the robot, subjects saw themselves as a robot. Tactile stimulation was provided by stroking synchronously or not with a paintbrush the same location of the subject and robot faces. In contrast to the previous motor induction of embodiment which particularly affected agency, tactile induction yields more generalized effects on the perception of ownership, location and agency. Interestingly, the links between positive social feelings towards the robot and the strength of the embodiment sensations were not observed. We conclude that the embodiment into a robot is not sufficient in itself to induce changes in social cognition.

## Introduction

Although humanoid robots provide new tools to investigate human social cognition^1–3^ understanding the underlying mechanisms of social human–robot interactions remains a challenge. Experimental methods from cognitive/social neuroscience constitute a reliable approach to study how we perceive and interact with robots. For example, multimodal (sensory and motor) manipulations during human–robot interactions can change the sense of embodiment (SoE), i.e. the perception of oneself as one body that belongs to me being able to integrate objects into one’s body-self representation^3–5^. The experience of SoE contributes to reveal the phenomenal self-representation also called minimal self that is the ‘I’ who is experiencing ‘now-here’^6^. In a key paper, Gallagher^6^ defines the minimal self as “a consciousness of oneself as an immediate subject of experience, unextended in time … depending on brain processes and an ecologically embedded body”. This concept of minimal-self considered as a momentary phenomenon is pertinent in the domain of robotics and has inspired robotic engineers for implementation of virtual minimal self into a robot design^6,7^.

The SoE is highly variable depending on the timing and the modality of the sensory-motor stimuli used to induce embodiment. As a whole, the SoE is regarded as having several underlying subcomponents including ownership (the sensation that an object or a body is part of my body) and agency (the sensation that I can exert control on an object or a body)^8–10^ as well as self-location (the sensation that I am located within an object or a body)^11^. These embodiment features have been revealed using a variety of experimental setups with explicit (e.g. questionnaire) and implicit (e.g. proprioceptive drift or physiological response) measures^12,13^. The rubber hand illusion (RHI) paradigm^14^ constitutes a pioneering experiment that revealed the ability of human participants to incorporate an artefact into their body representation hence producing a sensation of embodiment (SoE). Thus, watching a rubber hand being stroked synchronously with one’s own hidden hand induces the sensation that the rubber hand is part of one’s own body and one’s own hand is mislocalized towards the rubber hand^14–17^. This illusion does not occur when the rubber hand is stroked asynchronously with respect to the subject’s own hand. To date, inspired by the RHI paradigm, various attempts to examine the SoE with robotic devices have focused on certain body parts^18–20^ or exploited brain–computer interfaces^21^. The illusion of body ownership was successfully induced for robotic arms with a high resemblance to human arms in terms of shape^18^ and texture^22^, but also for non-human looking arms^20^. A sense of ownership can also concern an artificial face as Ma et al.^23^ created the virtual-face illusion by having participants control the movements of a virtual face in front of them. When the face moves in synchrony with their own face, the participants appear to perceive the virtual face as their own.

In contrast, fewer studies described a sense of body ownership for android robots moving in synchrony with the participant^24^. A first person perspective situation with congruent visuo-audio feedback effectively induces a sense of embodiment toward a humanoid robot while keeping a sense of location in the subject’s own body (illusion of bilocation)^25^. More interestingly, recent research showed that embodiment into a robot can yield changes in moral, affective and social thoughts. For example, Aymerich-Franch et al.^26^ investigated the experience of guilt during robot avatar embodiment where full-body ownership over a humanoid robot was induced using visuomotor correlations. Similarly, by using a simple neuroscience-inspired procedure to beam human subjects into a robot, Ventre-Dominey et al.^27^ investigated the sense of embodiment in relation with social attributes. Beaming via correlated (synchronous) vs. static or uncorrelated (asynchronous) human and robot head movements produced a strong effect of embodiment, including the sense of agency over the robot and a feeling of mislocation of the subject’s own body inside the robot. Moreover, subjects judged the robot that they embodied synchronously as more likeable and socially closer. In contrast, participants experienced no significant sense of enfacement in any beaming conditions. Similar to the ownership sensation over an artificial hand in the RHI experiment^14^, enfacement has often been described following sensory manipulations on the face^28–31^. Such an enfacement effect was reported for the first time by Tsakiris^28^ who showed that a multisensory integration of visuo-tactile facial stimuli influences the ability to distinguish one’s own face from the face of another person. Using a similar paradigm, Sforza et al.^29^ replicated this effect and demonstrated that the strength of the enfacement was positively correlated with the participants cognitive and emotional traits, hence suggesting that the sensation of enfacement may be modulated by social processing. Accordingly, Paladino et al.^32^ provided direct evidence of a social impact of multisensory stimulation of a subject’s face, as watching synchronous tactile stimulations on another’s face elicited more positive affective reactions and conformity toward the other than watching asynchronous stimulation. In human communication, the synchrony between behavioural events constitutes a key feature in the manifestation of the feeling to be embodied into the other, and also in self –recognition and social cohesion^29–33^. This observation has been extended to human–robot interactions as synchronous movements between human and robot can lead to more SoE^25,27^, acceptability^27^ and even anthropomorphisation towards the robot^34^.

Likewise, we recently described a link between the sense of embodiment, particularly the agency and location, induced by visuo-motor manipulation and social attractiveness toward a robot. In contrast to human face to face experiments, the social cognitive changes were independent of the occurrence of an enfacement sensation during visuo-motor interactions between human and robot. Several alternatives can explain such a discrepancy in the enfacement abilities between human–human and human–robot interactions: (1) the different visuo-tactile vs visuo-motor modalities of embodiment induction and (2) the different face to face and robotic telepresence setups. In the current study, we investigate the effects of sensory manipulations during robotic telepresence on the type (ownership, enfacement, location, agency) and the strength of embodiment and their relationship to social and affective attributes toward the robot. To address this issue, we used a telepresence paradigm similar to our previous experiment^27^ and we characterized subjects’ embodiment into a robot induced by visuotactile stimulation. By using a head-mounted visual display connected to the robot eyes, the subject sees the 3D visual environment of the robot and his/her own face as a robot face by placing a mirror in front of the robot. The sensation of embodiment was induced by simultaneously stroking the same locations on the subject and robot faces with a paintbrush. The synchrony effect was investigated by comparing correlated (synchronous) and uncorrelated (asynchronous) tactile stimulation conditions. We measured embodiment (Ownership, Enfacement, Location and Agency) scores and social acceptance (Likeability and Closeness) for the robot before and after the beaming experience, using standardized questionnaires. As in the previous study^27^, the experiment was performed with two robots distinct in their structures: the robot iCub, having a humanoid structure, and the robot Reeti resembling a cartoon character. By using two different robots, we could investigate synchronous and asynchronous conditions in each subject with a different robot, and whether robot embodiment would depend on the robot having or not a closer resemblance to the structure and morphology of the human body. Accordingly, in order to determine the respective effects—of the robot morphology, humanoid vs non-humanoid (R-Type) and—of the synchrony state, correlated versus uncorrelated (R-State), behavioral responses were compared between R-Type and R-State. These results reveal that tactile modalities can induce embodiment into a robot but the information transferred by the tactile modality appears to affect differently the SoE as compared to the motor modality as previously observed. Remarkably, though both modalities can induce a sense of agency, while voluntary head movements rendered a robot socially acceptable, passive tactile strokes on the cheek do not suffice to produce this acceptability.

## Results

In this section, we will first describe the results obtained in robotic telepresence with visuo-tactile manipulations. Second, we will compare these results with those obtained in our previous telepresence experiment using the same paradigm but with visuo-motor manipulations^27^.

### Effects of visuo-tactile manipulations

#### Embodiment score

**Figure 1:**
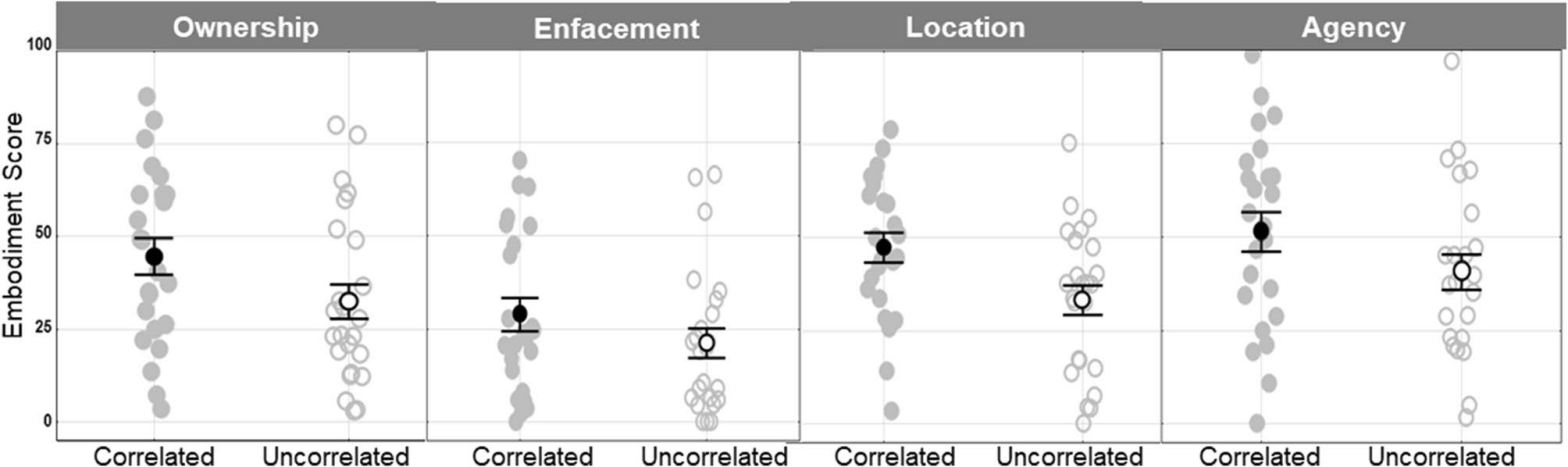
Mean values of the embodiment scores for the ownership, enfacement, location and agency categories as measured in the Correlated and Uncorrelated visuo-tactile stimulation conditions. Note the general increase in the scores in the Correlated as compared to Uncorrelated conditions. Grey points: individual data; Black circles: means; Bars: standard errors.

By using a one sample *t* test against the test value 0, the mean Embodiment score of each category (Ownership, Enfacement, Location, Agency) was significantly different from 0 in correlated and uncorrelated robotic states (R-States) (p < 0.001). While we observed an illusory sensation of embodiment in both states, the embodiment scores were significantly different between correlated and uncorrelated states (R-State effect *F*(1,22) = 17.23, *p* < 0.001, partial *η*^2^ = 0.439, α = 0.977) and between the embodiment categories (Category effect *F*(3,66) = 10.70, *p* < 0.001, partial *η*^2^ = 0.327, α = 0.998), with no significant interactions (R-State × Category interactions (*F*(3,66) = 1.27, *p* > 0.25*,* partial *η*^2^ = 0.054, α = 0.323). As shown in Fig. 1, the embodiment scores were significantly increased in the correlated as compared to the uncorrelated states independently of the embodiment categories. By post-hoc Bonferroni comparisons, we found significant differences between correlated and uncorrelated states for ownership, location and agency (p < 0.05), but not for enfacement (p > 0.25). Moreover, the averaged enfacement score was significantly reduced as compared to the averaged ownership (p = 0.003), location (p = 0.001) and agency (p < 0.0001) scores. In contrast, no significant difference was found between the averaged ownership, location and agency scores (p > 0.25). The mean scores of embodiment are reported for each statement of the different categories in the table of the supplementary material (SM Table). We did not find any significant effects of the Type of Robot (Reeti, iCub) (R-Type main effect: F(1,22) = 0.15, p > 0.25, partial η^2^ = 0.007, α = 0.07; R-Type × Category interactions: (F(3,66) = 0.59, p > 0.25; partial η^2^ = 0.026, α = 0.165).

#### Likeability measurement and IOS test

In contrast with the significant effects of correlated vs. uncorrelated visuo-tactile stimulation on embodiment described above, there was no significant effect of the visuo-tactile stimulation on likeability, nor on closeness tested by the Inclusion of Other in the Self (IOS).

Interestingly, we observed a significant effect of the R-Type factor on the Likeability score (*F*(1,22) = 8.44, *p* = 0.008; partial *η*^2^ = 0.28, α = 0.79) and less so on the IOS score (*F*(1,22) = 3.6, *p* = 0.071; partial *η*^2^ = 0.14, α = 0.44). As a whole, the robot Reeti was more likeable than the robot iCub. However, when analyzing the effects of R-State on the Likeability score independently for each robot, there was no significant difference between correlated and uncorrelated conditions neither for Reeti (R-state *F*(1,11) = 0.01, *p* > 0.25, partial *η*^2^ = 0, α = 0.05; R-Type × Beaming Experience interactions (F(1,11) = 0.77, p > 0.25; partial η^2^ = 0.065, α = 0.13) nor for iCub (R-state *F*(1,11) = 1.18, *p* > 0.25, partial *η*^2^ = 0.1, α = 0.17; R-Type × Beaming Experience interactions (F(1,11) = 1.39, p > 0.25; partial η^2^ = 0.11, α = 0.19). As a whole, the Likeability was not dependent on the R-State (F(1,23) = 0.33, p > 0.25; partial η^2^ = 0.04, α = 0.16) nor on the pre-post Beaming Experience (R-Stat × Beaming Experience interactions (F(1,23) = 0.28, p > 0.25; partial η^2^ = 0.012, α = 0.08). By computing the Bayes factor, the likelihood of the non-significant effect of R-State on the Likeability was supported (*t* test: t = 1.06, n1 = 24, n2 = 24, rscale = 0.707, Scaled JZS Bayes Factor = 2.20; Scaled-Information Bayes Factor = 1.63).

As concerns the feeling of Closeness, there was no significant effect of the R-State on the IOS score (R-State (F(1,22) = 1.43, p = 0.24; partial η^2^ = 0.061, α = 0.21). By computing the Bayes factor, the non-significance of R-State on the Closeness was supported (*t* test: t = 1.22, n1 = 24, n2 = 24, rscale = 0.707, Scaled JZS Bayes Factor = 1.90; Scaled-Information Bayes Factor = 1.39).

In summary, telepresence with visuo-tactile induction produces a significant embodiment effect for ownership, location and agency, but not enfacement. Visuo-tactile stimulation induces no effects for likeability nor inclusion of self in other.

### Comparison between visuo-tactile and visuo-motor manipulations

In this section, we compare the results obtained in the current visuo-tactile experiment to those previously obtained in the visuo-motor experiment using the same paradigm^27^. In the previous study, the embodiment was induced by visuo-motor interactions using correlated or uncorrelated head movements between a subject and a robot. Such a cross-modality statistical comparison will hence provide reliable clues about the impact of the sensory versus motor modalities on the embodiment into a robot as well as on the social attributes during robotic telepresence.

**Figure 2:**
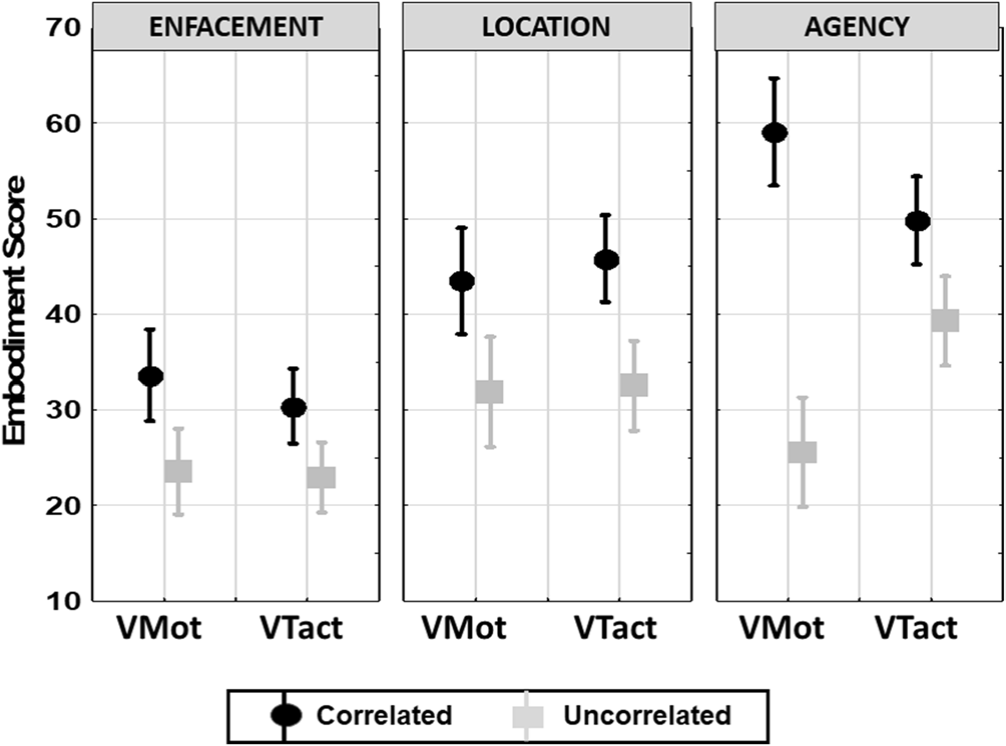
Mean values of the Agency Score as measured in the Correlated and Uncorrelated conditions in the two visuo-motor (VMot) and visuo-tactile (VTact) experiments. Circles: averaged agency scores; Bars: standard errors.

As the ownership was not evaluated in our previous experiment^27^, only the embodiment categories including enfacement, location and agency will be compared between visuo-motor and visuo-tactile modalities. As illustrated in Fig. 2, the effect of R-State on the embodiment scores was significantly dependent on the motor vs sensory modality and the category factors (R-State × Modality × Category interactions F(2,76) = 7.3, p = 0.0012; partial η^2^ = 0.16, α = 0.92). Interestingly, even though no specific significant effects were found by post-hoc Bonferroni analysis, Fig. 2 shows an important difference between the agency scores obtained with visuo-motor as compared to visuo-tactile modalities.

**Figure 3:**
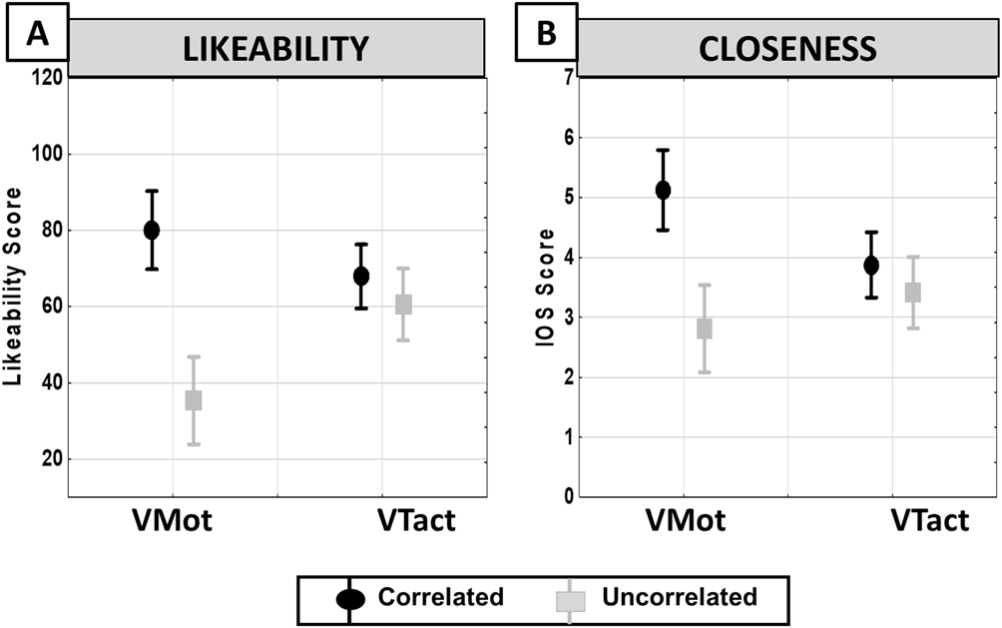
Mean values of the Likeability (**A**) and Closeness (**B**) scores as measured in the Correlated and Uncorrelated conditions in visuo-motor (VMot) and visuo-tactile (VTact) experiments. Circles: averaged scores of likeability and closeness; Bars: standard errors.

The social cognition components were significantly influenced by the sensory vs motor modalities. Likewise, the synchrony effect R-State on the Likeability scores was significantly dependent on the modality of the embodiment induction (R-State × Modality interactions F(1,38) = 11.20, p = 0.0018; partial η^2^ = 0.23, α = 0.90). As shown in Fig. 3A, the Likeability was significantly reduced in uncorrelated visuo-motor condition as compared to uncorrelated (p = 0.0032) and correlated (p < 0.001) visuo-tactile conditions. Similarly the closeness measured by IOS scores significantly differed in relation to both R-State and Modality factors (R-State × Modality interactions F(1,38) = 12.37, p = 0.0011; partial η^2^ = 0.25, α = 0.93). The IOS score was significantly increased in the correlated visuo-motor condition as compared to the correlated (p = 0.039) and uncorrelated (p = 0.0016) visuo-tactile conditions of the embodiment induction (Fig. 3B).

## Discussion

In this study we investigated the effects of tactile stimulation on the sensation of embodiment during robotic telepresence. In contrast to our previous findings relative to the perception of embodiment after visuo-motor manipulations^27^, the tactile induction of a robotic incorporation yields more distributed illusory perception in terms of ownership, location and agency, predominantly during correlated sensory stimulations. Interestingly, we thus observed a significant effect on agency with visuo-tactile stimulation in the absence of active visuo-motor stimulation. Moreover, the links between positive social feelings towards the robot, including the acceptability and the degree of the embodiment sensations were not observed.

Similarly to the setup of our previous robotic telepresence experiment^27^, human subjects wearing a head-mounted visual display saw the visual environment through robot eyes. Their sensation of incorporation into the robot was promoted by placing a mirror in front of the robot so that the subject sees him/herself as a robot. The sensation of embodiment was investigated by comparing correlated (synchronous) vs. uncorrelated (asynchronous) stroking of the same location of the subject and robot faces with a paintbrush. Subjects reported a feeling that the face of the robot was their own face (ownership), that they were at the place of the robot (location) and were able to control the robot movements (agency), significantly during correlated facial stroking. Interestingly, in our previous work, when the embodiment sensation was induced by visuo-motor cues, such as simultaneous head movements, the significant illusory effects were more restricted but more intense, particularly in terms of the sense of agency, i.e. the subjects reported a strong sensation of controlling the robot motor acts and this was predominantly observed during correlated movements^27^.

In the current study, the illusion of embodiment as measured by the strength of the sensation could be observed in both synchrony conditions, correlated and uncorrelated, even though the effects were always more pronounced when the sensory cues were synchronous between subject and robot. Here, the intensity of the sensation was measured on a subjective scale in a continuum between 0 (no sensation) and 100 (highly strong sensation) and thus could account for any low intensity of embodiment during correlated as well as uncorrelated stimulation. In contrast, a number of previous studies^9,14,35–38^ evaluated the embodiment score on the basis of an agreement level in response to each questionnaire statement using a Likert scale ranging from “− 3” (totally disagree) to “+ 3” (totally agree), with “0” indicating uncertainty. This disparity in the measurement methods of embodiment can be the source of discrepancy in embodiment results in uncorrelated stimulation between our positive values versus the null or negative values reported in the literature. Moreover, an alternative explanation of such embodiment scores in uncorrelated stimulations can also be related to our experimental setup using a mirror that reinforced a self-identification with the robot.

Based on our observations, we suggest that the sensation of embodiment into a robot is multifaceted and differs according to the multimodal manipulation employed. While the senses of ownership, location and agency were significantly impacted by our sensory manipulations, primarily the sense of agency was affected during motor manipulation using the same setup. Evidence has been provided that a sense of ownership and agency can rely on independent brain processes^9,10^. In this context, we can consider that the sense of agency observed in co-occurrence with a sense of ownership during our current visuo-tactile manipulation relies on a different neurocognitive process than the sense of agency observed during the previous visuo-motor manipulation. Indeed, the sense of agency itself is not unitary and is made up of several dimensions^9,10,39^. Comparing RHI experiments using active and passive motor cues versus visuotactile cues, Kalckert et al.^9,39^ demonstrated that the sense of agency is dissociated into two components: (1) external agency related to the voluntary motor command to external objects that is a goal-directed agency independent of the anatomical body congruency and (2) body agency, closely related to the sense of ownership observed during active movements but highly dependent on the anatomical body congruency and thus encoded in ego-centered coordinates in contrast to the external agency. In line with Longo’s observations^11^, Kalckert et al.^9,39^ also noted a sense of agency in the passive movements and visuotactile conditions when the visuosomatic feedback occurred synchronously compared to asynchronously. The authors suggest that even in the absence of an engaged agency mechanism, the presence of ownership drives a residual sense of (body) agency. Interestingly, the relative contribution of the motor vs sensory systems on the sense of ownership and agency has also been investigated in the context of cross-modal stimulation by Kokkinara and Slater^13^. The authors demonstrated that while visuomotor correlation is more powerful than visuotactile in establishing general embodiment, this effect is broken to the same extent by incongruences in either visuomotor or visuotactile stimulation. These observations from the literature and our own research are suggestive of independent but interacting brain processes responsible for the sensation of a body agency associated to ownership as compared to the sensation of agency driven by the intentional motor processes.

The hypothesis of two distinct processes generating embodiment phenomenology is largely supported by the neurocognitive models describing the sense of agency (SoA) and the sense of ownership (SoO). While the visuo-tactile induction provides passive afferent information coming from the external world, the motor induction relies on voluntary motor command linked to internally generated intentional processes. In this context, Braun et al.^10^ review several internal state models explaining the functional dissociations of the embodiment phenomenology depending on the sensory or motor modality of the induction. Based on RHI studies, the sense of ownership was first accounted for by bottom-up processes related to Bayesian perceptual learning of multisensory afferent correlations, including spatio-temporal correlations between visual and tactile stimulations^40^. Likewise, evidence has been provided for the existence of a top-down process including one or several comparators that evaluate the fitness between the afferent sensory information and a pre-existing body model^41–43^. Only a match resulting from the comparator will give rise to a sensation of ownership and location. In contrast, the sense of agency would be linked to high-order intentional processing as described by the classical comparator model of motor control^44,45^. Accordingly, an internal feedforward comparator compares two signals: -one as a prediction of the sensory outcomes of the action computed as an efference copy of the motor command and—the other as the external sensory feedback which informs about the real outcomes of the movement. Thereby, the motor error resulting from this comparison allows to distinguish between externally and self-produced events and thus to inform the sense of agency. If predicted and estimated actual signals are congruent, the sensory event is attributed to our own action and gives rise to the own agency. If not, the causality of the action is attributed to another agent with no sense of agency on the sensory event^46–49^.

At this point of the discussion, it is important to refer to more cognitive domains that link the high-order form of agency to conceptual processes like the authorship of an action, the contextual and environmental beliefs^46–49^. In particular Synofzik et al.^46,47^ suggest theoretical links between agency and belief-like process, background contexts and social rules which parallel our observation on the correlation between the sense of agency and the feeling of likeability and closeness toward a robot. The Likeability and Closeness were investigated and compared between the two robots, iCub and Reeti. While the Closeness was determined with the well-described Inclusion of Other in Self test (IOS)^50^, the Likeability was established through an original test using subjective scale comparable to that described by Monohan et al.^51^ in their human communication studies. In a literature review Bartneck et al.^52^ gave emphasis to several interesting social indicators including Likeability towards robots as measured by Monohan’s^51^ reliable questionnaire (Chronbach’s Alpha > 0.7) with 5 items of liking judgements using a 5-point semantic differential scale (1 = not at all to 5 = quite a bit)*.* Similarly, we have evaluated the feeling of Likeability on one item (“how likeable is the robot for you?”) on a subjective scale between 0 (not Likeable at all) to 100 (very much likeable). Interestingly, in our robotic telepresence experiments, changes in social traits including both likeability and closeness were linked to embodiment only during the visuo-motor^27^ and not during the visuo-tactile induction of the illusory sensations. What are the critical features of the sensory and motor induced embodiment that produce such a beneficial change in social attraction in our robotic telepresence experiments? Indeed, neither the occurrence nor the scores of embodiment per se are sufficient to induce a social benefit. It is noteworthy to recall that in our experiment the subject was embodied into a robot facing a mirror and thus was seeing him/herself as a robot. Thus, seeing the robot’s face being touched or moving at the same time as one’s own face likely evoked changes in the mental representation of the subject’s face shifting the self-other boundary as previously reported^30,32,53,54^. In human psychology, the role of synchrony in mutual movements has been demonstrated to be determinant in the perception of social similarity and the interpersonal relationship^33^. These observations have also been paralleled and extended to human–robot interactions as synchronized head movements between robot and human can induce acceptability^27^ and anthropomorphism^34^ towards the robot. In recent robotic investigations^55,56^, self-recognition abilities have been built in humanoid robots placed in front of a mirror using visual features coupled to kinesthetic cues. Through algorithms based on a theoretical model of perception and action in the brain and neural network learning, the robot was able to perform self-recognition in a mirror using the mirror self-recognition test and distinguish its own simple actions from others. In these studies^55,56^ basic self-recognition in the robots was grounded on combined sensory- motor synchrony and more importantly, by exploiting the prediction error.

Based on these findings, we suggest that the determinant cue that induces social changes in combination with illusory body sensations resides in the intentional causality of the experimental embodiment phenomenology. In other words, it is likely that the intentional command of the simultaneous movements is crucial to produce a change in the social feeling co-occurring with the sensation of embodiment into the robot. This argument is supported by the concept of the mirror neuron system that implements a resonance mechanism in the primate brain, allowing for shared action representations to be established between interacting agents^57–59^. In this context, a number of studies suggest an implication of the mirror neuron system in higher-order functions beyond action representation, including intention understanding^60,61^, empathy, theory of mind^62–64^, and self-recognition^65^. In our embodiment experiments, the activation of the mirror neuron system reflecting an intentional resonance during head movements simultaneously between a human subject and a humanoid robot might induce a social binding not observed during a pure shared visuo-tactile stimulation.

## Conclusion

Visuo-tactile induction of a robotic incorporation yields an illusory perception of embodiment in terms of ownership, location and interestingly agency, predominantly during correlated sensory stimulations. In contrast to our previous findings relative to the perception of embodiment after visuo-motor manipulations^27^, there were no changes in social cognitive features after visuo-tactile interactions that were observed after visuo-motor interactions. These results are suggestive of dissociated mechanisms underlying embodiment particularly in terms of agency in correlation with social feelings toward robots. We suggest that the intentional resonance evoked during correlated movements between human and robot are responsible for an increased feeling of social and emotional proximity toward a robot.

## Material and methods

The material and methods are based on the same paradigm, setup and data analysis as those described in the previous paper^27^.

### Material

#### Participants

Twenty-four healthy right-handed volunteers (mean age = 24.0 years, SD = 4.3) participated in the experiment. A power analysis (G*Power software-version 3.1.9.3 software) revealed that the subject numerosity was greater (0.81) than the one required assuming a standard power value of 0.8. In order to ensure optimal objectivity, participants were unaware of the specific aim of the study. They gave their informed consent prior to the experiment. The study was approved (Rhône-Alpes Préfecture: Authorization No. 10028) by the Regional Health Agency (Agence Régionale de Santé-ARS) review board authorizing biomedical research at the Stem Cell and Brain Research Institute where the experiments were carried out in accordance with the principles of the revised Helsinki Declaration (World Medical Association, 2013).

#### Robot description

This study was performed with two robots: the robot iCub^66^ (Italian Institute of Technology, Genoa, Italy) and the robot Reeti (Robopec, 83140 Six-Fours-les-Plages France, http://www.reeti.fr/index.php/en/). Briefly, concerning the overall appearance, the iCub robot has a humanoid structure, approximately the size of a 3-year-old child, while the robot Reeti resembles a cartoon character. Both are expressive robots with animated faces thanks to mobile eye and heads with numerous degrees of freedom. They are equipped with video cameras in the eyes allowing the robot to explore the visual environment.

### Methods of the visuo-tactile experiment

#### Experimental setup

**Figure 4:**
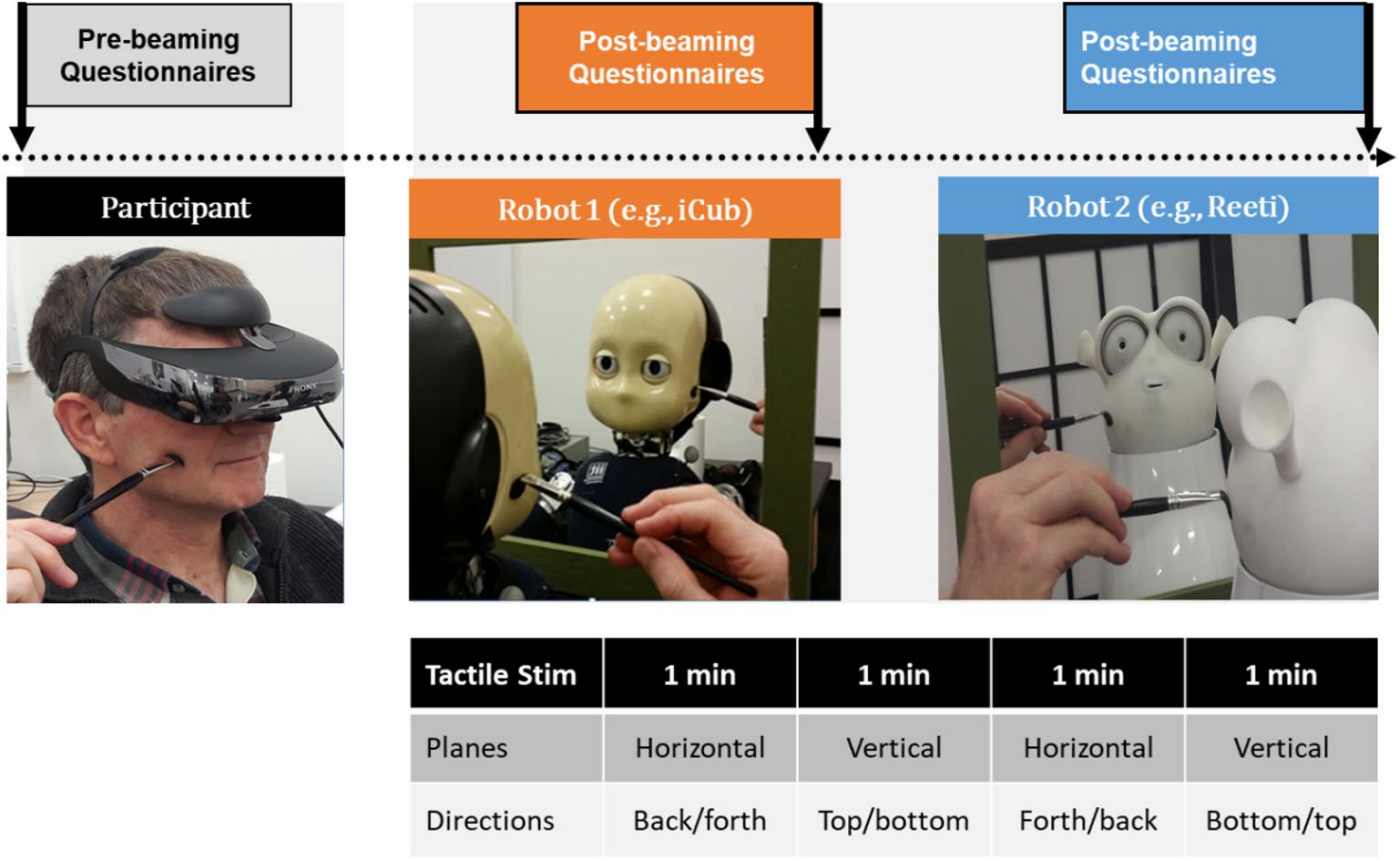
The robot setup illustrating (left) the human subject with head mounted display being stimulated on the right cheek, (middle) the iCub facing the mirror, stimulated on the right cheek, (right) the Reeti robot in front of the mirror, stimulated on the left cheek. Timeline indicates Pre-beaming questionnaires followed by Robot 1, Post-beaming questionnaires, then Robot 2, Post-beaming questionnaires. The panel (bottom) gives the successive 1 min phases of visuo-tactile stimulations realising one correlated or uncorrelated session of robotic telepresence. Informed Consent to publish the participant images has been obtained from study participant Jocelyne Ventre-Dominey.

Subjects sat in the experimental room in a location out of the robot’s visual field in order not to be visible by the robot and thus not to see themselves through the eyes of the robot. As shown in Fig. 4, the subjects wore a stereo Head-Mounted Display (HMD) (SONY HMZ-3WT 3D Viewer) connected to the video cameras located in the robot eyes. This setup immediately teleported the subject into the robot’s visual environment. An audio headset isolated the subject from environmental noises to favor the virtual immersion. The immersive teleoperation in the robot was reinforced by a mirror, positioned on a mobile table, placed at a distance of 20 cm, in front and at the height of the robot’s face. In consequence, the subjects saw themselves in the mirror as a robot.

The sensory manipulation was induced by tactile stimulation using a paintbrush stroking the same location of the subject and robot cheeks. As illustrated in Fig. 4, two trained experimenters delivered manually the tactile stimulations—one experimenter was sitting next to the subject so that the subject only felt the moving paintbrush and—the other one next to the robot. A large computer screen visible only to the experimenters displayed a moving target that indicates the starting point, the direction (horizontal or vertical) and the frequency of the tactile stimulation. The target (red circle) started from one corner of the screen and moved in the horizontal or vertical direction. Both experimenters started to stroke the subject and robot faces at the exact time of the target onset and maintained the paintbrush displacement synchronous to the target motion at a frequency of 0.33 Hz. In the correlated condition the two experimenters were moving the paintbrush synchronously to the visual target and in the uncorrelated condition one experimenter was displacing the paintbrush with a delay of about one second with respect to the other experimenter. The regions of the robot face to be stroked were delimited by marks only visible to the experimenter in order to stimulate the facial zones most corresponding to those of the subject. The whole setup was positioned in order for the experimenter stimulating the subject to visually monitor the correlation or not of the tactile stimuli (paintbrush movements) applied to subject’s and robot’s faces. Prior to the experimental session, both experimenters were trained to respect the spatio-temporal constraints of tactile stimulations.

#### Procedure

The successive steps of the visuo-tactile procedure are illustrated in Fig. 4.

##### Pre-beaming phase

In order to have an optimal effect, subjects were told that “the study is aimed to determine the best psychological profile for a robot to be further involved in social interactions in commercial or cultural contexts”. Then the subject was introduced to each of the robots (names, examples of movements) and he/she was asked to complete the questionnaire on Likeability as described below.

##### Beaming phase

Once the beaming set-up was completed, including the accurate positioning of the mirror in front of the robot, the subject saw the robot’s face as well as the robot head and shoulders in place of his/her own face and body reflected in the mirror. Subjects were instructed to maintain their gaze on the robot’s face reflected in the mirror.

The two experimenters were located laterally behind the subject and the robot respectively, and repeatedly stroked the subject and robot cheeks with identical paintbrushes. Two sequences of tactile stimuli were used, respectively, in the horizontal direction with the paintbrush moving horizontally back and forth on the lower jaw or in the vertical direction between the cheek bone and the jaw along the plane of the eyes. Half of the subjects were stimulated on the right and half on the left cheek.

The beaming phase consisted of two successive experimental sessions of robotic teleoperation with each robot respectively. During one experimental session the tactile stimulations were presented in four alternated sequences of horizontal and vertical paintbrush strokes starting from the front or the back of the jaw for horizontal movements and from the top or the bottom of the cheek for vertical movements. The horizontal and vertical sequences and their respective starting points were counterbalanced over the sessions and the subjects. (Bottom panel in Fig. 4).

Central to this interaction was the synchrony feature of the tactile stimulation: either synchronous (correlated session) or asynchronous (uncorrelated session) between subject and robot. Each subject underwent two experimental sessions that differed in the timing of the tactile stimulations either (1) correlated, with the paintbrush strokes synchronous between subject and robot or (2) uncorrelated, with the paintbrush strokes asynchronous i.e. with a 1 s delay introduced between the paintbrush moves of the subject and those of the robot. Thus, in the uncorrelated session the temporo-spatial congruency between the tactile sensation felt on the subject’s own face and the tactile stimulus seen on robot’s face was disrupted.

After the first beaming session, the subject responded to the questionnaires (Inclusion of the Other in the Self, Embodiment statements). Then, the subject was tested with the second robot with the opposite tactile stimulation synchrony. Hence, the first robot could be experienced in the correlated tactile stimulation condition, whereas the second robot was experienced in the uncorrelated tactile stimulation condition (or vice-versa). The order of the robot type (iCub or Reeti), and of the temporal conditions (correlated or uncorrelated) were counterbalanced across participants.

##### Post-beaming phase

At the end of the beaming sessions with the two successive robots, the subject was asked to respond to the questionnaire of Likeability towards the two robots, then during a debriefing time to describe his/her sensations induced by the experimental beaming into the robots and the visuo-tactile stimulations.

#### Questionnaires and dependent variables

One questionnaire was used to quantify the degree of embodiment into the robots and two questionnaires were used to quantify the degree of social acceptance toward each robot i.e. the Likeability and the Closeness measured with the Inclusion of the Other in the Self test (IOS). For each questionnaire, the subject was asked to rate between the two extremes of a subjective scale his/her sensation during the telepresence session.

##### Embodiment test

Subjective reports about the sense of embodiment were collected by asking participants to fill out a questionnaire that was adapted from the first, seminal study on the rubber hand illusion^14^. The participant rated her/his degree of embodiment into the robot on a subjective scale ranging from 0 (no sensation of embodiment) to 100 (highly strong sensation of embodiment). This embodiment questionnaire was presented to the participant after each experimental session (correlated and uncorrelated). Based on the Gonzalez-Franco & Peck’s^38^ review, the questionnaire is made up of four types of questions each composed of several items presented to the subject:

- the feeling that the robot’s body belongs to the own body (*Ownership*: 3 items),
  *1—I had the feeling that I was seeing myself in the mirror.*
  *2—I had the feeling that the robot's head was part of my body.*
  *3—I had the feeling that the robot's head was my own head.*
- the feeling to resemble the robot’s face (*Enfacement*: 5 items),
  *4—I had the feeling that my face started to resemble the robot's face.*
  *5—I had the feeling that the robot’s face started to resemble my face.*
  *6—I had the feeling that my skin became pale.*
  *8—I had the feeling that my nose was smaller.*
  *9—I had the feeling that my skin became rubbery.*
- the feeling to be at the location of the robot’s body (Location: 4 items),
  *10—I had the feeling that I was in the place of the robot.*
  *11—I had the feeling that my position alternated between my own body and the robot's body.*
  *12—I had the feeling that the touch I felt on my own face was due to the paintbrush touching the robot's face.*
  *13—I had the feeling that I was transported inside the robot.*
- the feeling to control the robot’s motion (Agency: 6 items).
  *14—I had the feeling that I could control the robot's head.*
  *15—I had the feeling that I lost control of my own face.*
  *16—I had the feeling that if I made a face, the robot would make the same face*
  *17—I had the feeling that I could control the robot's eyes.*
  *18—I had the feeling that the movements of the robot head could reproduce my own movements.*
  *19—I wanted to move as a robot.*

##### Measurement of likeability

Subject’s likeability for the robot was estimated twice—once after the robot presentation and before the first beaming session (Pre-beaming test) and—the other after both beaming sessions at the end of the experiment (Post-beaming test). The subject evaluated her/his degree of Likeability for the robot on a subjective scale rated from 0 (not at all likeable) to 100 (very likeable) and displayed below the robot picture. This same test was presented to the subject in the Pre- and Post-beaming phases of the experiment.

##### Self-other closeness task

**Figure 5:**
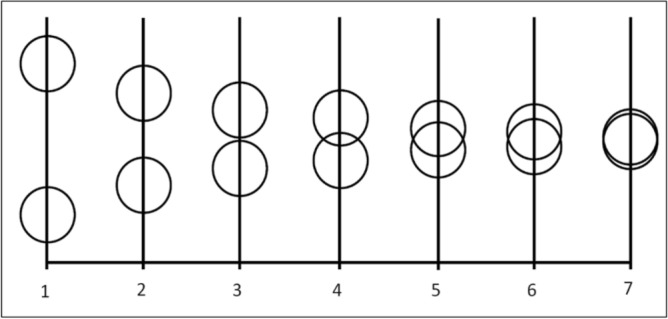
The Self-Other Closeness task as an adaptation of the single-item Inclusion of Other in the Self (IOS) scale developed by Aron et al.^50^. The pairwise circles illustrate the self (here the participant) and the other (here the robot). On the bottom, the subjective scale of the level of Closeness.

The Self-Other Closeness task is an adaptation of the single-item Inclusion of Other in the Self (IOS) scale developed by Aron et al.^50^ to measure how close the respondent feels with another person or group. This task aims to evaluate the degree of closeness toward the robot. A graphic 7-point scale was made of a series of two coupled circles illustrating the self (here the participant) and the other (here the robot) with different degrees of overlapping from distant circles (1) to coinciding circles (7) (see Fig. 5). Thus, participants see seven pairs of circles that range from distant to almost completely overlapping and choose one of the seven pairs which best matches the degree of closeness feeling to the robot. This test was presented after each experimental beaming session (i.e., correlated, uncorrelated).

### Methods for the visuo-motor experiments

In the visuo-motor experiment as described in our previous paper^27^, a group of 16 healthy, right-handed subjects (8 males, mean age = 23.80. years SD = 4.00) participated in the experiment. All of the subjects reported no history of drug abuse, nor neurological nor psychiatric disorders. In this earlier experiment, the robots and the set-up were comparable to the current visuo-tactile experiment with the subject seeing her/himself as a robot through a mirror. As a main distinction, a head position sensor (Polhemus Fastrack, 05446 Vermont, USA) fixed on the HMD display could register the signal of head motion that was transmitted to the robot via a PC computer. With this set-up called Super Wizard of Oz (SWoOZ)^67,68^, the robot could reproduce the subject’s head displacements with a delay not detectable by the subject. In the visuo-motor condition, the procedure was composed of the same steps as in the visuo-tactile experiment with a pre-beaming, a beaming and a post-beaming phase. The only difference relied on the modality used to induce telepresence i.e. the visuo-motor modality realised by simultaneous head movements between the robot and the subject. Thus in the beaming phase, the subject performed two sessions of telepresence: with one robot, a session of correlated head movements (Correlated session) where the robot’s head movements could be synchronous to the subject’s head movements and with the other robot, a session of uncorrelated head movements (Uncorrelated session) where the robot’s movements were independent of the subject’s movements. The same tests of Likeability and Closeness were used to evaluate the social features. To measure the scores of embodiment, three categories of sensations -enfacement, location and agency-were measured in each Correlated and Uncorrelated conditions.

### Data analysis

Statistical analyses were performed with Statistica software package (TIBCO Software Inc., CA 94304, USA). All variables were initially analysed with the Kolmogorov–Smirnov method to test for the data normal distribution and with the Levene test to probe the homogeneity of variances. As all the variables distribution met the assumptions of normality and variance homogeneity at p > 0.05, we used statistical parametric tests, including the analysis of variance (ANOVA) for Likeability, Closeness and Embodiment scores.

For Likeability, we used a mixed repeated measures ANOVA design with two within-subject factors as Beaming Experience (Pre, Post) and Robot State (R-State: Correlated, Uncorrelated) and one between subject factor Robot Type, identifying which robot was experienced (R-Type: Reeti, iCub). For the IOS score, a mixed repeated measures ANOVA with one within-subject factor, the Robot State (R-State: Correlated, Uncorrelated) and one between subject factor, the Robot Type (R-Type: Reeti, iCub). For the Embodiment scores, a one sample *t* test to the zero referent value was initially used to evaluate whether the mean of the Embodiment score at each category was statistically different from the null value. Then, a mixed repeated-measures ANOVA was used with two within-subject factors, the Robot State (R-State: Correlated, Uncorrelated) and the Embodiment Category (Ownership, Enfacement, Location, Agency) and the between-subject factor the Robot Type (R-Type: Reeti, iCub). Post-hoc specific pairwise comparisons were realised with a Bonferroni test and the significance was established at 95% of confidence interval. Finally, the comparison between the visuo-motor and visuo-tactile experiments was performed on each variable that is the Likeability, Closeness (IOS test) and Embodiment scores using a mixed rmANOVA using two within-subject factors, the Robot State (R-State: Correlated, Uncorrelated) and the Embodiment Category (Enfacement, Location, Agency) and an additional between-subject factor, the Modality of the stimulation (Motor,Tactile). To establish the likelihood of non-significant results, we calculated the Bayes factors based on Bayesian t test calculation from Rouder et al.^69^ (Bayes Factor Package-http://pcl.missouri.edu/bayesfactor).

## Supplementary Information

Supplementary Table 1.
