# Supplementary material for: Insights on embodiment induced by visuo-tactile stimulation during robotic telepresence
Source: Sci Rep. 2021 Nov 22;11:22718. doi: 10.1038/s41598-021-02091-8 (PMC8609005; doi:10.1038/s41598-021-02091-8)
Supplement: Supplementary file 1 — Supplementary Table 1. [file 41598_2021_2091_MOESM1_ESM.docx]

| **EMBODIMENT** | ***STATEMENTS*** | **CORR** | **UNCORR** |
| --- | --- | --- | --- |
| **OWNERSHIP** | *I had the feeling that I was looking at myself in the mirror* | 57.1 (30) | 37.6 (29) |
|  | *I had the feeling that the robot's head was part of my body* | 40.2 (33) | 27.5 (26) |
|  | *I had the feeling that the robot's head was my own head* | 36.5 (24) | 32.4 (28) |
|  | **Total statements for ownership** | 44.5 (24) | 32.5 (23) |
|  |  |  |  |
| **ENFACEMENT** | *I had the feeling that my face started to resemble the robot's face* | 44.5 (29) | 30.9 (29) |
|  | *I had the feeling that the robot’s face started to resemble my face* | 21.3 (24) | 12.7 (10) |
|  | *I had the feeling that my skin became pale* | 27.1 (27) | 22.2 (27) |
|  | *I had the feeling that my nose was smaller* | 27.6 (32) | 23.9 (30) |
|  | *I had the feeling that my skin became rubbery* | 23.3 (28) | 17.4 (24) |
|  | **Total statements for enfacement** | 28.8 (22) | 21.2 (20) |
|  |  |  |  |
| **LOCATION** | *I had the feeling that I was in the place of the robot* | 56.3 (29) | 38.7 (26) |
|  | *I had the feeling that my position alternated between my own body and the robot's body* | 30.7 (29) | 29.0 (30) |
|  | *I had the feeling that the touch I felt on my own face was due to the paintbrush touching the robot's face* | 63.4 (30) | 33.5 (28) |
|  | *I had the feeling that I was transported inside the robot* | 38.6 (28) | 31.5 (27) |
|  | **Total statements for location** | 47.2 (19) | 33.2 (20) |
|  |  |  |  |
| **AGENCY** | *I had the feeling that I could control the robot's head* | 48.3 (33) | 35.9 (32) |
|  | *I had the feeling that I lost control of my face* | 48.1 (31) | 38.3 (26) |
|  | *I had the feeling that if I made a face, the robot would make the same face* | 48.7 (31) | 37.2 (30) |
|  | *I had the feeling that I could control the robot's eyes* | 51.2 (29) | 45.4 (32) |
|  | *I had the feeling that the movements of the robot head could reproduce my own movements* | 51.2 (32) | 37.2 (30) |
|  | *I wanted to move as a robot* | 61.1 (34) | 52.8 (34) |
|  | **Total statements for agency** | 51.5 (26) | 41.0 (23) |

SM Table of embodiment scoring by statement comparing correlated (CORR) vs uncorrelated (UNCORR) conditions of visuo-tactile stimulation.
